# The pathophysiological role of MiRNAs in heart failure

**DOI:** 10.3389/fcvm.2026.1753834

**Published:** 2026-06-03

**Authors:** Kun Lian, Qing Qi, Lichong Meng, Xin Zhu, Yubin Zhang, Yichang Xu, Zhiguang Song, Lin Li, Siyuan Hu, Zhixi Hu

**Affiliations:** Hunan University of Chinese Medicine, Changsha, China

**Keywords:** heart failure, mechanism of action, miRNAs, myocardial fibrosis, precision diagnosis

## Abstract

**Background:**

Heart failure (HF) is a growing global health issue, chiefly marked by inexorable myocyte loss, maladaptive remodeling, and energetic failure. MicroRNAs (miRNAs) function as the principal post-transcriptional regulators of gene networks capable of facilitating the pathophysiology of illnesses. However, there is limited systemic-level understanding of their participation in human HF. Therefore, the miRNA mechanisms that modulate apoptosis, autophagy, myocardial fibrosis, inflammatory response, and energy metabolism of cardiomyocytes to inhibit and treat HF are elucidated in this work.

**Method:**

A analysis of the mechanisms utilized by miRNAs in the modulation of HF was performed following the retrieval of literature on the mechanism of action of miRNAs in HF. The literature was obtained from PubMed and Web of Science databases between 2021 and 2025.

**Result:**

The multi-dimensional regulatory function of miRNAs in HF was uncovered. At the cellular fate level, they mediate the survival and death of the myocardium and inhibit excessive apoptosis and oxidative damage. At the organizational structure level, they inhibit the advancement of pathological ventricular remodeling and fibrosis. At the energy homeostasis level, they control autophagy and energy metabolism of cells. Finally, at the inflammatory and signaling levels, they suppress the inflammatory storm and maintain calcium signaling homeostasis. A sophisticated and dynamic regulatory system consisting of various miRNAs was observed, which offers a theoretical basis and potential targets for HF mechanism research and targeted intervention.

**Conclusion:**

MicroRNAs (miRNAs) jointly affect the survival, remodeling and energy balance of cardiac muscle cells. However, the clinical application of miRNAs in HF is still in the early exploration stage, and their true diagnostic value and therapeutic potential need to be further confirmed by rigorous and large-scale clinical studies.

## 1 Introduction

Heart failure (HF) is a clinical condition identified by symptoms resulting from structural and functional cardiac abnormalities, marked by raised levels of natriuretic peptides and objective indicators of pulmonary or systemic congestion (1). Due to its high prevalence, high mortality, and high expenses, this condition has grown to be an international health issue (2). In recent years, young adults (aged 15–44 years) saw a rise in age-adjusted mortality from 2.36 in 1999 to 3.16 in 2019, compared to older adults (aged ≥75 years) (3). Moreover, HF risk factors comprise advancing age, gender, hypertension, hereditary cardiomyopathy, diabetes mellitus, and obesity. Understanding these factors facilitates the significance of implementing targeted prevention strategies (3).

Several potential aetiologies, specific risk factors, and co-morbidities participate in HF progression; however, the mechanisms associated with these factors in the development and advancement of HF remain under investigation (4). Novel diagnostic tools and advances in therapeutic approaches have had a substantial impact on the diagnosis and treatment of this condition, leading to enhanced prognosis and life expectancy. Nevertheless, hospitalization and mortality rates continue to be high (5).

The microRNAs (miRNAs) are small endogenous RNA molecules of approximately 22 nucleotides identified in 1993. They function as negative regulators of the messenger RNA (mRNA) and regulate post-transcriptional expression of genes, affecting the majority of physiological and pathological processes (6). These miRNAs are differentiated by their small size, relative stability, and significant influence on the regulation of gene expression (7). During the miRNA generation, the miRNA is transcribed in the nucleus, then carried to the cytoplasm, where it is divided into two mature strands. One strand binds to the cytoplasm to function as a negative regulator of gene expression, while the other strand undergoes degradation (8). These molecules serve a variety of crucial roles at the cellular process regulation level, including cell growth and differentiation, apoptosis, and tumour progression (9). Consequently, the dysregulation of miRNA contributes to the onset of numerous diseases.

Recent research has demonstrated that miRNAs are pivotal in the development and advancement of several conditions, including HF (10–14). These results have encouraged further investigation into the potential of circulating miRNAs as HF biomarkers and therapeutic agents. Therefore, the impact and mechanism of action of miRNA in HF were examined in this work, with the aim of offering reference and guidance for the prevention and management of HF. However, taking into account the timeliness and length of the article, this paper selects the relevant literature published from 2021 to 2025.

## 2 Biogenesis and release of miRNA

Biogenesis and release of miRNAs in mammalian cells happen through tightly controlled processes that transform primary transcripts into mature, functional RNAs. This process can be broadly divided into nuclear and cytoplasmic phases, and it may follow either canonical or non-canonical pathways depending on the miRNA and cellular conditions (15).

### 2.1 Canonical miRNA biogenesis and release

RNA Polymerase II transcribes miRNA genes into long primary transcripts (pri-miRNAs) containing hairpin structures (15). In the nucleus, the Microprocessor complex (Drosha and its cofactor DGCR8) cleaves the pri-miRNA to produce a pre-miRNA, a shorter hairpin structure ∼60–70 nt long (16). Exportin-5 (XPO5) exports the pre-miRNA from the nucleus to the cytoplasm through a process that requires the Ran-GTP-bound state (17). The RNase III enzyme Dicer cleaves the pre-miRNA close to the terminal loop in the cytoplasm with the help of its cofactor TRBP, producing a ∼22 nt miRNA duplex (18). One of the double-stranded strands (the guide strand) is bound to the Argonaute (Ago) protein, forming an RNA-induced silencing complex (RISC). The complementary passenger strand is discarded (15, 19). Through seed pairing (nucleotides 2–8), the mature miRNA directs RISC to complementary target mRNAs, resulting in translational repression and/or mRNA degradation (20).

### 2.2 Non-canonical pathways

Certain miRNAs deviate from the canonical biogenesis pathway by bypassing key enzymes: mirtrons are spliced from introns and directly form pre-miRNAs without the need for Drosha cleavage, whereas miR-451 is processed by Drosha but is subsequently cleaved by Ago2 instead of Dicer to generate the mature miRNA (21–23).

### 2.3 Release and turnover

**Figure 1:**
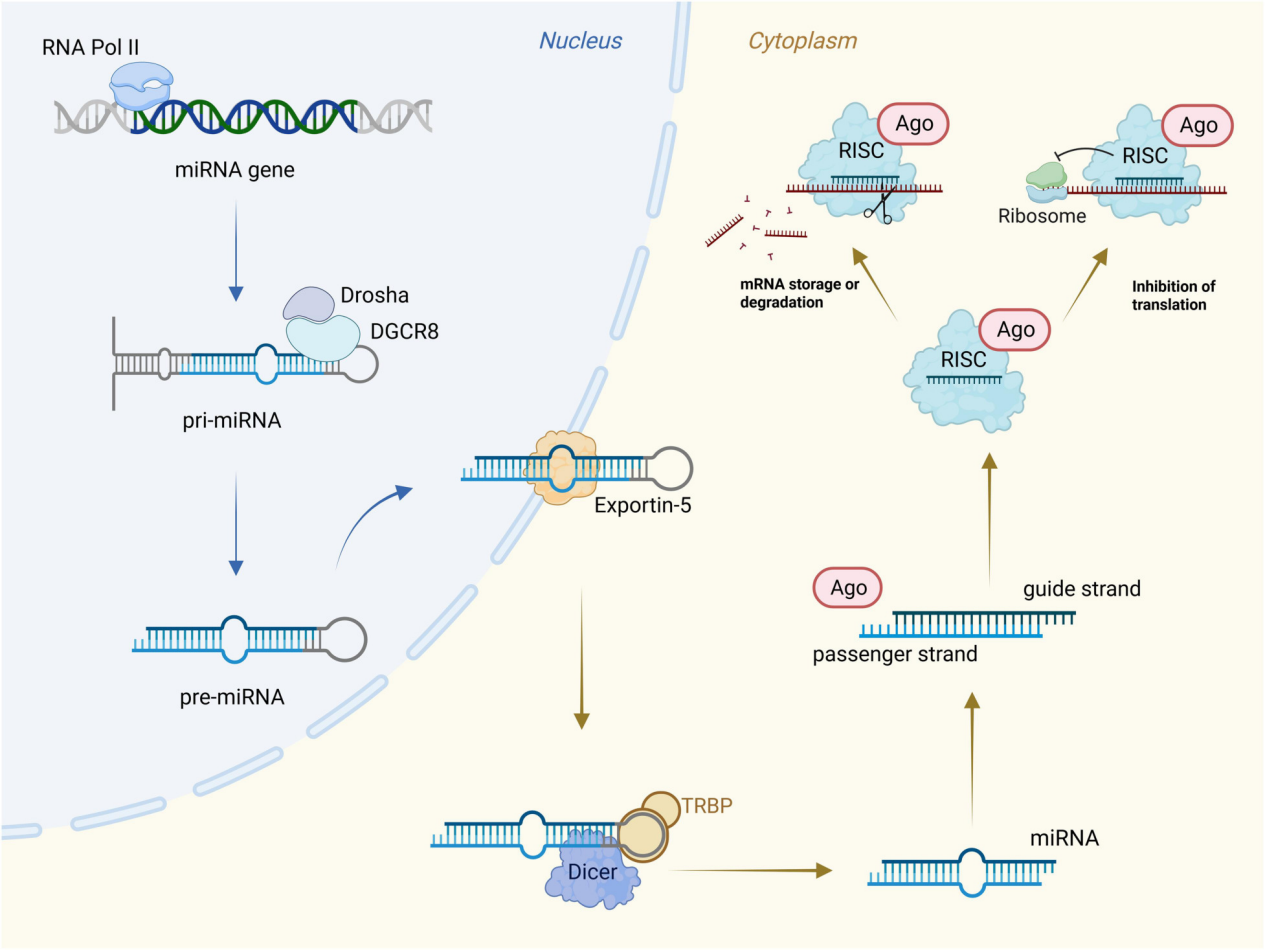
Biosynthesis and release of miRNAs. (Generated by BioRender.com).

Although the vast majority of mature miRNAs are retained inside cells as stable, functional complexes with Ago proteins, a measurable fraction is continually exported to the extracellular space (24). The miRNAs that were secreted can be found encapsulated within 30–150 nm exosomes, residing in larger microvesicles that bud directly from the plasma membrane, or simply hitch-hike on soluble RNA-binding proteins like Ago2, nucleophosmin-1, or high-density lipoprotein (HDL) particles (25–30). The extracellular miRNA pool that results from this release appears to be cell-type and stimulus-specific, and it is currently widely used as diagnostic “liquid-biopsy” markers (31). Regardless of their location, intracellular miRNAs are not inert; their half-lives can be actively reset by a conserved quality-control pathway termed target-directed miRNA degradation (TDMD). When an RNA exhibits significant complementarity to an Ago2-bound miRNA, the resulting complex undergoes a conformational change that allows the ZSWIM8 E3 ubiquitin ligase to reveal and ubiquitinate specific surface lysines on Ago2. Ubiquitylated Ago2 is then routed to the proteasome, releasing the bound miRNA for rapid 3′ → 5′ exonucleolytic digestion (32, 33). Thus, both export and TDMD cooperate to shape the steady-state miRNA repertoire inside and outside the cell. The biosynthesis and release of miRNAs are shown in Figure 1.

## 3 Mechanism of miRNA regulation of heart failure

**Figure 2:**
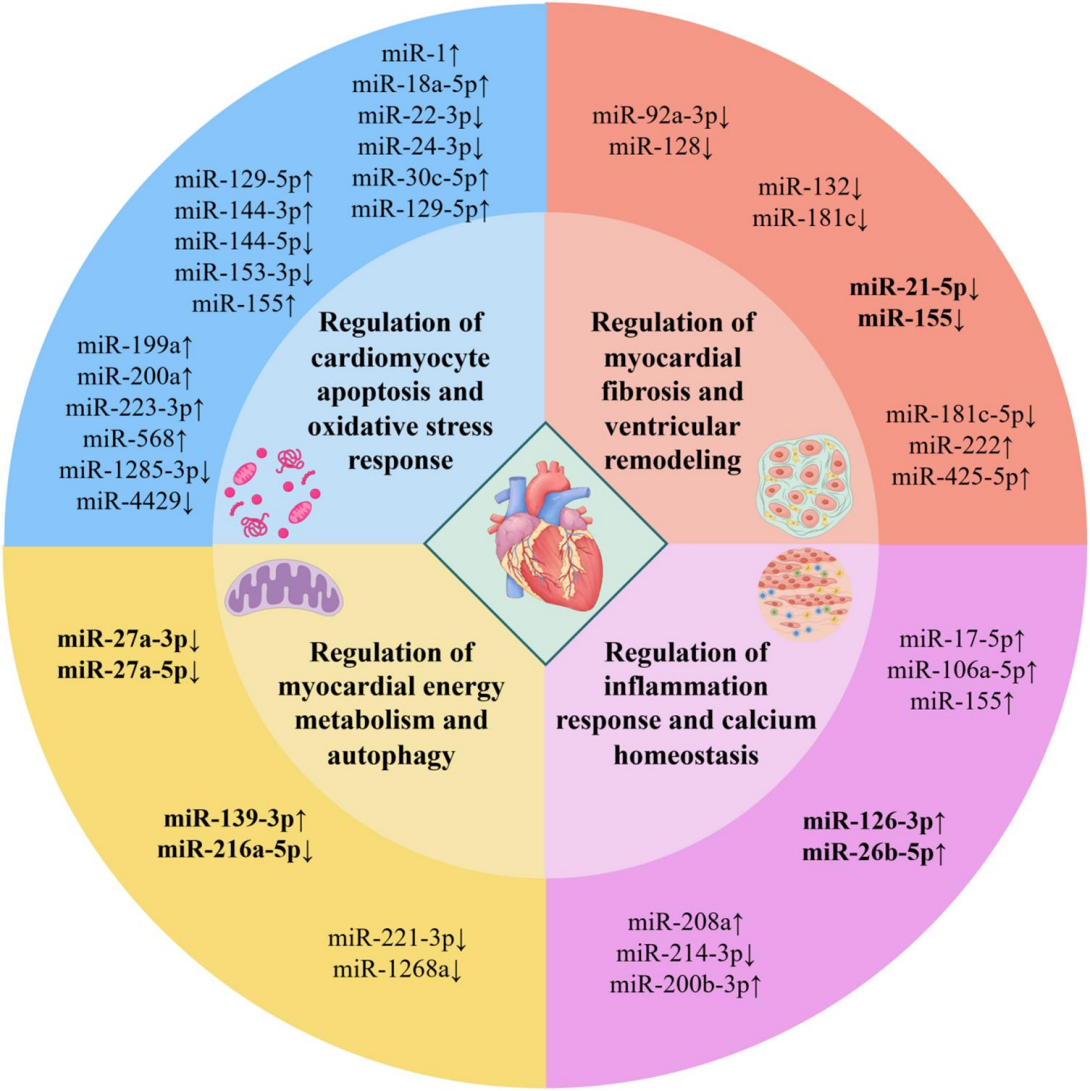
MiRNAs affect the mechanism of action of HF. (Generated by figdraw.com). The arrows indicate the changes in miRNA expression between the heart failure group and the healthy control group, while the bold font and arrows represent the changes in miRNA expression between the drug intervention group and the heart failure group.

MiRNA is an endogenous non-coding RNA that can develop a “multi-target, cross-cell” regulatory matrix among endothelial cells, fibroblasts, cardiomyocytes, and inflammatory cells. It controls key pathological connections like myocardial hypertrophy, fibrosis, inflammatory response, and apoptosis by blocking or degrading target mRNA (34, 35). We can surmise that a comprehensive knowledge of the mechanism by which miRNA controls chronic heart failure will help address the limitations of traditional single-target therapy and offer a new theoretical basis and transformation path for the targeted therapy of chronic heart failure. Several topics, like the regulation of miRNAs on cardiomyocyte apoptosis, oxidative stress response, myocardial fibrosis, ventricular remodeling, myocardial energy metabolism, and inflammatory response, were addressed in this work, as outlined in Figure 2.

### 3.1 Regulation of cardiomyocyte apoptosis and oxidative stress response

Damage to cardiomyocytes is a form of pathological hallmark of HF (36). In cardiac cells, HF induces mitochondrial dysfunction in cardiac cells, s, leading to a disruption of the electron transport chain. This then results in the increased production of reactive oxygen species (ROS), which aggravates cellular damage, and causes mitochondrial contents, including factors that induce apoptosis, to leak out and initiate apoptotic cell death (37). Apoptosis elevates the incidence and progression of HF by reducing cardiomyocytes and myocardial contractility, thus highlighting the critical participation of apoptosis in HF (38, 39). Down-regulated cardiac pro-mitochondrial biosynthetic factors facilitate the decline in mitochondrial oxidative phosphorylation and a reduced ability to oxidize fatty acids, which impairs myocardial energy production and further accelerates HF (40).

Oxidative stress occurs during an imbalance between the generation of ROS and their elimination. This process is a key factor in the pathophysiology of HF and cardiac remodeling (41). The adverse effects of ROS in HF include the activation of signaling pathways linked to apoptosis, the proliferation of cardiac fibroblasts, mtDNA damage, impaired mitochondrial function, and cardiac hypertrophy (42–44). From clinical and experimental research, it has been demonstrated that the levels of oxidative stress in the myocardium and at the systemic level are elevated during HF (45, 46). Therefore, the modulation of oxidative stress-mediated signaling pathways can enable the prevention or delay of the onset of HF (47).

Based on research, miRNAs participate in the regulation of the oxidative stress response and myocardial cell apoptosis, delaying the onset of HF. It was discovered that miR-1 was considerably reduced using the H9c2 cell model of oxygen-glucose deprivation/reoxygenation (OGD/R). Overexpression of miR-1 can target and inhibit the HCN2/HCN4 pathway. Lower TNF-α and IL-6 levels improve cell viability and reduce apoptosis (48). Doxorubicin (DOX) administration to establish a chronic heart failure (CHF) rat model revealed a reduction in the expression of miR-18a-5p. MiR-18a-5p overexpression can target and inhibit Notch2, lower Cleaved-caspase-3 and Bcl-2-associated X protein (Bax) levels. This overexpression also results in lower Notch2, Hes1, and Hes5 mRNA and protein expressions, increased Bcl-2 protein expression, and reduced cardiomyocyte apoptosis (49). The expression of miR-22-3p in the HF rat model was observed to increase. Thus, inhibiting miR-22-3p can target and restore FURIN, reduce serum BNP levels, and inhibit cardiomyocyte apoptosis (50).

Further research has demonstrated the substantial elevation of miR-24-3p in the HF rat model. The inhibition of miR-24-3p can target and activate the Sp1/PI3K pathway, significantly lower N-terminal pro b-type natriuretic peptide (NT-proBNP), caspase-3, lactate dehydrogenase (LDH), and ROS levels, suppress myocardial cell apoptosis and oxidative stress, and strengthen cardiac function and tissue structure (51). The serum from patients with CHF was analyzed by XIONG (52), who observed a down-regulation in the expression of miR-30c-5p. Additional cell experiments demonstrated that the overexpression of miR-30c-5p effectively suppressed apoptosis in HUVECs while also enhancing their proliferation, migration, and invasion. When AC16 (human cardiomyocyte cell line) cells were treated with H_2_O_2_, miR-129-5p decreased. The increased expression of miR-129-5p prohibits apoptosis and autophagy by lowering ATG7 (an essential protein involved in autophagy) (53). Research has demonstrated that in the CHF rat model induced through ligation of the left anterior descending coronary artery, miR-129-5p was reduced. Raising miR-129-5p targets Smurf1 and blocks its action, leading to the restoration of PTEN expression. This modulation results in decreased Malondialdehyde (MDA) levels, increased superoxide dismutase (SOD) and glutathione peroxidase (GSH-Px) levels, thereby alleviating oxidative stress (54).

MiR-144-3p was substantially decreased in the DOX-induced HF rat model. The increase of miR-144-3p can target and inhibit cytokine signal transduction inhibitor 2 (SOCS2), significantly increase Bcl-2 and p-PI3K/Akt levels, reduce the expressions of Bax and Cleaved-caspase-3, inhibit cardiomyocyte apoptosis, and improve cardiac activity and histopathological damage (55). Kao et al. (56) confirmed that miR-144-5p levels rose substantially in the DOX-induced HF mouse model. Blocking miR-144-5p can target and restore the expression of ACSM1, leading to a notable reduction in biomarkers like BNP, LDH, Ang II, and ALD, inhibiting lipid peroxidation and apoptosis, and thus improving left ventricular function and histopathological changes. AC-16 and HCM cells treated with H_2_O_2_ experienced a down-regulation of MIR17HG and SIRT1, but an up-regulation of miR-153-3p. Inhibiting miR-153-3p can restore SIRT1 expression, significantly reduce ROS and NPPB levels, and improve oxidative stress (57).

In an HF mouse model induced by aortic arch constriction (AAC), Luo et al. (58) discovered that myocardial tissue had markedly lower levels of miR-155. However, following the administration of miR-155 agonists, there was an improvement in cardiac function, a notable reduction in the expression of apoptosis-related proteins Bax and Cleaved-caspase-3, an increase in Bcl-2, and a suppression of cardiomyocyte apoptosis by targeting HIF-1α. In the coronary ligation-induced HF rat model, miR-199a nanoparticles were administered for intervention, and it was found that they could substantially lower the expression of HSP27 and SGP130, raise the level of HSP70, and inhibit cardiomyocyte apoptosis and inflammatory response (59). Studies on patients with CHF and a CHF rat model prepared by ligation of the left anterior descending coronary artery showed a notable drop in miR-200a levels. However, overexpression of miR-200a can target and inhibit HMGB1, significantly reduce interleukin-1 (IL-1), tumor necrosis factor-α (TNF-α), BNP, MDA, and CK-MB levels, inhibit myocardial cell apoptosis and oxidative stress, and improve myocardial tissue structure (60).

The overexpression of miR-223-3p in H9C2 cells treated with OGD can lower levels of IL-6, IL-1β, and Cleaved-Caspase-3, preventing inflammation and apoptosis (61). Researchers developed a rat model for CHF by narrowing the aorta. In this model, the levels of miR-568 dropped substantially. Overexpression of miR-568 can target and inhibit SMURF2, significantly reduce the levels of serum LDH, AST, CK, and CK-MB, alleviate myocardial cell apoptosis and oxidative stress, and improve cardiac function and histopathological damage (14). A study by ZHANG (62) illustrated through clinical research and cell experiments that inhibiting miR-1285-3p enhanced cell growth and angiogenesis, while lowering cell apoptosis.

Cell experiments confirmed that miR-4429 was significantly elevated in cardiomyocytes induced by H_2_O_2_. Consequently, inhibiting miR-4429 can target and restore the expression of HAPLN1, reduce the level of ROS, inhibit oxidative damage, and improve cell viability and myocardial structure (63).

MiRNAs effectively regulate the survival and death of cardiomyocytes by targeting apoptosis and oxidative stress-related indicators. Specific miRNAs such as miR-1, miR-18a-5p, miR-30c-5p, miR-129-5p, miR-223-3p, and miR-568 can inhibit pro-apoptotic pathways and enhance antioxidant defense. This dual action contributes to a reduction in myocardial injury and helps to slow the progression of HF, positioning these miRNAs as promising molecular targets for the prevention and management of this condition. On the other hand, miR-22-3p, miR-24-3p, miR-144-5p, miR-153-3p, and miR-4429 may aggravate cardiomyocyte apoptosis or oxidative stress response. By preventing their overexpression, the oxidative stress response and myocardial cell apoptosis are improved, and the advancement of HF is slowed. Table 1 summarizes the mechanisms and targets of miRNA for modulating oxidative stress responses and apoptosis (14, 48–63).

**Table 1:** The mechanism and targets of miRNA in regulating apoptosis and oxidative stress response.

Models and intervention methods	miRNAs	Regulatory indicators	Mechanism of action	References
OGD/R induces H9c2 cells	miR-1↑	TNF-α, IL-6↓	Target HCN2/HCN4 to enhance cell viability and reduce cell apoptosis	(48)
The CHF rat model was established by injecting DOX	miR-18a-5p↑	C-caspase-3, Bax, Notch2, Hes1, Hes5↓	Target Notch2 to inhibit cardiomyocyte apoptosis	(49)
		Bcl-2↑		
The HF rat model was established by injecting DOX	miR-22-3p↓	FURIN↑	Inhibit the apoptosis of myocardial cells	(50)
DOX-induced HF rat model	miR-24-3p↓	NT-proBNP, Caspase-3, LDH, ROS↓	Target Sp1 to inhibit apoptosis and oxidative stress	(51)
CHF patients, HUVECs cells	miR-30c-5p↑	BNP↓	Inhibit apoptosis and promote cell proliferation, migration, and invasion	(52)
H_2_O_2_ induces AC16 cells	miR-129-5p↑	ATG7↓	Improve apoptosis and autophagy	(53)
The rat model of CHF was established by ligation of the left anterior descending coronary artery	miR-129-5p↑	MDA↓	Target Smurf1 to reduce oxidative stress	(54)
		PTEN, SOD, GSH-Px↑		
DOX-induced HF rat model	miR-144-3p↑	Bax, C-caspase-3↓	Target SOCS2 and inhibit apoptosis	(55)
		Bcl-2, p-PI3K/AKT↑		
DOX-induced HF mouse model	miR-144-5p↓	BNP, LDH, AngII, ALD↓	Target ACSM1 to inhibit lipid peroxidation and apoptosis	(56)
H_2_O_2_ induces AC-16 and HCM cells	miR-153-3p↓	ROS, NPPB↓	Target SIRT1 to improve oxidative stress	(57)
		SIRT1↑		
The HF mouse model was prepared by the transverse contraction of the abdominal aorta	miR-155↑	Bax, C-caspase-3↓	Target HIF-1α to inhibit cardiomyocyte apoptosis	(58)
		Bcl-2↑		
Coronary ligation was used to prepare the HF rat model	miR-199a↑	HSP27, SGP130↓	Inhibit myocardial cell apoptosis and inflammatory responses	(59)
		HSP70↑		
The rat model of CHF was established by ligation of the left anterior descending coronary artery	miR-200a↑	HMGB1, IL-1, TNF-α, BNP, MDA, CK-MB↓	Target HMGB1 to inhibit cardiomyocyte apoptosis and oxidative stress	(60)
OGD induces H9C2 cells	miR-223-3p↑	IL-6, IL-1β, Cleaved-Caspase-3↓	Target MAPK to inhibit apoptosis and inflammation	(61)
The CHF rat model was established by the coarctation of the aorta	miR-568↑	LDH, AST, CK, CK-MB↓	Target and inhibit SMURF2 to alleviate apoptosis and oxidative stress	(14)
HUVECs cells	miR-1285-3p↓	Cell proliferation and angiogenesis↑	Reduce apoptosis	(62)
CHF patients and H_2_O_2_-induced cells	miR-4429↓	ROS↓	Target HAPLN1 to inhibit oxidative stress	(63)
		HAPLN1↑		

OGD/R, Oxygen-Glucose Deprivation/Reperfusion, TNF-α, Tumor Necrosis Factor-alpha, IL-6, Interleukin-6, CHF, Chronic heart failure, DOX, Doxorubicin, Bax, Bcl-2-associated X protein, Bcl-2, B-cell lymphoma-2, HF, Heart Failure, LDH, Lactate Dehydrogenase, ROS, Reactive Oxygen Species, BNP, B-type Natriuretic Peptide, MDA, Malondialdehyde, SOD, Superoxide Dismutase, GSH-Px, Glutathione Peroxidase, ALD, Aldosterone, HMGB1, High Mobility Group Box 1, CK-MB, Creatine Kinase-MB Isoenzyme, AST, Aspartate Transaminase, CK, Creatine Kinase.

### 3.2 Regulation of myocardial fibrosis and ventricular remodelling

Cardiac fibrosis occurs during an excessive deposition of collagen fibers in the heart muscles. This occurs in injured hearts, and extensive and sustained fibrosis impairs heart function (64, 65). This process is highly influenced by the origin and stage of the disease; thus, it is heterogeneous and dynamic (66, 67). This event is a defining feature of HF and other heart disorders and represents multifaceted health complications that have been challenging for effective therapeutic interventions (68). Activated cardiac fibroblasts (CFs), which trigger the pathological deposition of excess extracellular matrix (ECM) proteins within the perivascular and interstitial cardiac spaces, are the chief mediators of cardiac fibrosis (69). The fibroblasts migrate to the site of injury, proliferate locally, and eventually transdifferentiate into myofibroblasts (70). ECM molecules like collagens and fibronectin are primarily produced by activated cardiac fibroblasts (71, 72). Cardiac fibrosis is characterized by both the quantity and quality of collagen.

HF is triggered by pathological cardiac remodelling, which influences the clinical outcomes of this condition significantly. This process occurs through several complex mechanisms that result in alterations like pathological cardiac hypertrophy, interstitial fibrosis, higher degradation of the myocardial extracellular matrix, impaired heart functions, and even HF (73–76). During ventricular remodelling, the ventricular structure is altered, with changes such as shifts in the wall thickness and cavity size. Furthermore, the ventricle also moves from an elliptical form to a more spherical one. Myocardial hypertrophy is a common feature of cardiac remodeling in HF (77). Moreover, it can lead to a decline in cardiac function, particularly affecting systolic performance (78, 79).

We have found that many miRNAs can improve myocardial fibrosis and ventricular remodelling, effectively delaying the development process of HF. CHEN (80) found that miR-21-5p levels rose substantially and SKP2 expression fell in the DoX-induced CHF rat model. Paeonol intervention significantly suppressed themiR-21-5p, restored the level of SKP2, reduced the levels of BNP, LDH, Ang II, ROS, and ALD, decreased the expressions of Bax, c-caspase-3, and cytochrome C in the cytoplasm, and inhibited the apoptosis and fibrosis of myocardial cells.

A constructed HFpEF mouse model utilizing a high-fat diet and n-nitro-L-arginine methyl ester revealed that miR-92a-3p was increased. Blocking miR-92a-3p lowers COL1A1, COL3A1, FN1, IL-6, and TNF-α, and alleviates diastolic dysfunction and left atrial enlargement (81). In another investigation involving a CHF mouse model induced with isoproterenol (ISO), miR-128 was observed to rise. Thus, blocking miR-128 may target and activate MDFI, inhibit the Wnt1/β-catenin pathway, and alleviate myocardial hypertrophy and apoptosis (82).

By injecting ISO or TAC to induce HF mouse models, the miR-128 levels were upregulated. Inhibition of miR-128 can target and activate Axin1, suppress the Wnt/β-catenin pathway, reduce Wnt1 and β-catenin levels, and alleviate myocardial hypertrophy (83). Wu et al. (84) established an HFpEF mouse model through a high-fat diet and N-nitro-L-arginine methyl ester, and then administered an anti-miR-132 intervention. It was found that it could significantly inhibit the expression of P-Smad3 in the myocardium, reduce TGF-β 1-induced collagen synthesis and Smad3 phosphorylation, and lower the degree of myocardial hypertrophy and fibrosis. Research indicates (85) that the expression of miR-155 induced in ISO-induced CHF mouse models is suppressed by Schisandra chinensis. It further suppresses the phosphorylation of the AKT/CREB pathway, lowers the levels of ANP, BNP, β-MHC, and α-SMA, and mitigates myocardial cell hypertrophy and fibrosis. In older patients with HFpEF and diabetes, levels of miR-181c in the blood rose markedly. *In vitro* experiments revealed that miR-181c targets PRKN and SMAD7 to block them, alleviating myocardial fibrosis (86).

Boen et al. (87) constructed an HF mouse model by combining a high-fat diet with Ang II and L-NG-nitroarginine methyl ester. Their research revealed a significant increase in miR-181c-5p levels within the myocardium. Furthermore, inhibiting miR-181c-5p can target and reduce the expression of TGF-βRI in the heart, significantly reducing collagen deposition and myocardial fibrosis.

Using TAC, a stress-stressed HF mouse model was developed, and it was discovered that miR-222 expression rose. Overexpression of miR-222 can target and inhibit NFATc3, PUMA, and HMBOX1, significantly reduce the expressions of COL3A1, MMP-2, Caspase-1, Caspase-3, ANP, BNP, and β-MHC, suppress pathological myocardial hypertrophy, and improve left ventricular ejection fraction and survival rate (88). The investigation also revealed that miR-425-5p was notably downregulated in cardiac fibroblasts of this HF mouse model. The construction of a fibroblast-specific miR-425-5p overexpression model led to targeted inhibition of the TGF-β1/Smad signalling pathway, significantly suppressing the proliferation and differentiation of CFs, lowering the expression of FN, CTGF, Col1a1, Col3a1, α-SMA, and PCNA, and alleviating myocardial fibrosis, myocardial hypertrophy and contractile dysfunction (89).

In conclusion, by reducing the expression of proteins like COL1A1, COL3A1, and α-SMA, miR-222 and miR-425-5p can prevent myocardial fibrosis and ventricular remodeling, thereby alleviating HF. However, miR-21-5p, miR-92a-3p, miR-128, miR-128, miR-132, miR-155, miR-181c, and miR-181c-5p may exacerbate myocardial fibrosis and ventricular remodelling. Myocardial fibrosis can be alleviated and HF relieved through the downregulation of these miRNAs. We summarized the mechanisms and targets of miRNA in regulating myocardial fibrosis and ventricular remodeling, as shown in Table 2 (80–89).

**Table 2:** The mechanism and targets of miRNA in regulating myocardial fibrosis and ventricular remodeling.

Models and intervention methods	miRNAs	Regulatory indicators	Mechanism of action	References
DOX-induced CHF rat model, treated with paeonol	**miR-21-5p↓**	BNP, LDH, AngII, ROS, ALD, Bax, C-Caspase-3↓	Target SKP2 to inhibit apoptosis and fibrosis	(80)
		SKP2↑		
The HFpEF mouse model was prepared by a high-fat diet and N-nitro-L-arginine methyl ester	miR-92a-3p↓	COL1A1, COL3A1, FN1, IL-6, TNF-α↓	Improve diastolic function and left atrial enlargement	(81)
The CHF mouse model was prepared by injecting ISO	miR-128↓	Wnt1/β-catenin↓	Target MDFI to alleviate myocardial hypertrophy and apoptosis	(82)
		MDFI↑		
The HF mouse model was prepared by injecting ISO or TAC	miR-128↓	Wnt, β-catenin↓	Target and activate Axin1 to alleviate myocardial hypertrophy	(83)
The HFpEF mouse model was prepared by a high-fat diet and N-nitro-L-arginine methyl ester	miR-132↓	P-Smad3, collagen synthesis↓	Target Smad3 to alleviate myocardial fibrosis and myocardial hypertrophy	(84)
The CHF mouse model was prepared by ISO and treated with Schisandra chinensis methyl	**miR-155↓**	ANP, BNP, β-MHC, α-SMA↓	Inhibit the AKT/CREB pathway to alleviate myocardial cell hypertrophy and fibrosis	(85)
Adult cardiac fibroblasts	miR-181c↓	PRKN, SMAD7↑	Target PRKN and SMAD7 to alleviate myocardial fibrosis	(86)
The HF mouse model was established by a high-fat diet combined with Ang II and L-NG-nitroarginine methyl ester	miR-181c-5p↓	TGF-βRI↓	Targeting TGF-βRI in the heart, anti-fibrosis	(87)
TAC was used to prepare the HF mouse model	miR-222↑	COL3A1, MMP-2, Caspase-1, Caspase-3, ANP, BNP, β-MHC↓	Target NFATc3, PUMA, and HMBOX1 to inhibit pathological myocardial hypertrophy	(88)
TAC mouse model	miR-425-5p↑	FN, CTGF, COL1A1, COL3A1, α-SMA, PCNA↓	Target TGF-β1/Smad to alleviate myocardial fibrosis and myocardial hypertrophy	(89)

DOX, Doxorubicin, BNP, B-type Natriuretic Peptide, LDH, Lactate Dehydrogenase, ROS, Reactive Oxygen Species, ALD, Aldosterone, Bax, Bcl-2-associated X protein, HFpEF, Heart Failure with preserved Ejection Fraction, COL1A1, Collagen Type I Alpha 1 Chain, IL-6, nterleukin-6, TNF-α, Tumor Necrosis Factor-alpha, CHF, Chronic Heart Failure, ISO, Isoproterenol, TAC, Transverse Aortic Constriction, ANP, Atrial Natriuretic Peptide, β-MHC, Beta-Myosin Heavy Chain, α-SMA, Alpha-Smooth Muscle Actin, FN, Fibronectin, CTGF, Connective Tissue Growth Factor.

The markers of miRNAs that are regulated through drug intervention are marked in bold.

### 3.3 Regulation of myocardial energy metabolism and autophagy

Cardiomyocytes require an oxygen supply and key nutrients like glucose, fatty acids (FAs), and ketone bodies for ATP generation. This energy is pivotal for the maintenance of systolic and diastolic functions (90, 91). Alteration in energy metabolism is crucial to HF, leading to an “energy deficit” that exacerbates the condition's severity (92).Significant changes in energy substrate utilization and overall metabolic profile occur in the failing heart, which can result in both an imbalanced cardiac energy metabolism and a general reduction in ATP generation (93). This impaired energy generation stems from several factors, including disrupted mitochondrial metabolism, alterations in the heart's energy substrate preference, and reduced cardiac efficiency (94). Disturbances in these metabolic pathways that produce ATP can severely impact heart function (93). Therefore, a major factor in the severity of HF is impaired cardiac energy generation (95). Repairing substrate homeostasis has been shown to improve cardiac function in systolic dysfunctional mice (96). In addition, improving the metabolic profile of the heart and/or upgrading cardiac energy metabolism are promising therapeutic approaches for HF (93).

In eukaryotic organisms, autophagy or autophagocytosis is a key degradative process (97). Ideal autophagic activity promotes heart repair after injury by removing damaged organelles, reducing the production of free radicals, and maintaining intracellular stability (98). On the other hand, overactivation of autophagy may accelerate the development of HF by triggering cardiomyocyte death via autophagy-related pathways (99, 100). In late stages of HF, autophagy often gets blocked, most likely due to cellular metabolic exhaustion, which hinders the function of the autophagy pathway (101). Disrupted autophagy speeds up remodelling of the ventricular and deteriorates cardiac function by impeding the removal of harmful substances (101).

Modern research indicates that miRNAs can optimize cardiac function and promote the improvement of HF by regulating myocardial energy metabolism and autophagy. In the HF rat model was constructed by TAC, administration of Huangqi Danshen Decoction (HDD) significantly inhibited the release of miR-27a-3p in exosomes of pericardial adipose tissue, targeted and restored the expression of AMPKα2, and activated the PINK1/Parkin-mediated mitochondrial autophagy pathway. These actions significantly improved myocardial energy metabolism, reduced mitochondrial damage, and inflammatory infiltration (102). Xu et al. (103) used Ang II-induced AC16 cells and found that miR-1268a was significantly upregulated. Consequently, the inhibition of miR-1268a can target and restore the expression of CD36, significantly enhance ATP generation, uptake of fatty acid, and mitochondrial respiratory function, reduce the levels of BNP and ST2, and regulate myocardial energy metabolism and oxidative stress.

An established CHF and HUA rat model through a series of specific interventions, including intraperitoneal injection of DOX, intragastric administration of ethambutol, and subcutaneous injection of potassium oxazinate, revealed a reduced expression of miR-27a-5p and an increased expression of miR-139-3p following the administration of Huashui Jiangzhuo formula. It was further discovered that the AMPK/mTOR pathway was activated, the expression of p-AMPK protein reduced, and the expression of p-mTOR protein increased. Autophagy in cardiomyocytes was promoted with improved cardiac function (104). Literature has confirmed that ginsenoside Rb2 can significantly reduce the expression of miR-216a-5p in the HF rat model prepared by ligation of the left anterior descending coronary artery, increase the activities of Bcl-2, LC3B II/I, Beclin1, SOD, and CAT, and inhibit the levels of Bax, Caspase-3, MDA, and ROS. Consequently, ginsenoside Rb2 enhances autophagy, reduces apoptosis, and oxidative stress (105). Cao and Guo (106) induced an HF mouse model with DOX and found that METTL14 was significantly upregulated. Thus, the knockout of METTL14 was observed to suppress miR-221-3p levels, restore the levels of LncRNA FTX and SESN2, significantly reduce CK, CK-MB, LDH, NLRP3, GSDMD-N, IL-1β, and IL-18, and regulate pyroptosis and energy metabolism of myocardial cells.

In summary, some microRNAs may affect key molecules in the energy metabolism and autophagy pathways. However, there are relatively few miRNAs that help improve energy metabolism and autophagy. As shown in Table 3 (102–106), we summarized the mechanisms and targets of miRNA regulation of myocardial energy metabolism and autophagy.

**Table 3:** The mechanism and targets of miRNA in regulating myocardial energy metabolism and autophagy.

Models and intervention methods	miRNAs	Regulatory indicators	Mechanism of action	References
The HF rat model was prepared by TAC and treated with Huangqi Danshen Decoction	**miR-27a-3p↓**	AMPKα2, PINK1, Parkin↑	Target AMPKα2 to improve myocardial energy metabolism and mitochondrial damage	(102)
AngII induces AC16 cells	miR-1268a↓	BNP, ST2↓	Target CD36 to regulate myocardial energy metabolism and oxidative stress	(103)
		ATP↑		
The rat model of CHF complicated with hyperuricemia was established by intraperitoneal injection of DOX, intragastric administration of ethambutamol, and subcutaneous injection of potassium oxazinate. Huashijiangzhuo Decoction was used for treatment	**miR-27a-5p↓**	p-AMPK↓	Activate the AMPK/mTOR pathway to promote autophagy in cardiomyocytes	(104)
	**miR-139-3p↑**	p-mTOR↑		
The HF rat model prepared by ligation of the left anterior descending coronary artery was treated with ginsenoside Rb2	**miR-216a-5p↓**	Bax, Caspase-3, MDA, ROS↓	Enhance autophagy, reduce apoptosis, and oxidative stress	(105)
		Bcl-2, LC3BII/I, Beclin1, SOD, CAT↑		
DOX-induced HF mouse model	miR-221-3p↓	CK, CK-MB, LDH, NLRP3, GSDMD-N, IL-1β, IL-18↓	Regulate pyroptosis and energy metabolism of myocardial cells	(106)
		SESN2↑		

HF, heart failure, TAC, transverse aortic constriction, BNP, B-type natriuretic peptide, ST2, suppression of tumorigenicity 2, ATP, adenosine triphosphate, CHF, chronic heart failure, DOX, doxorubicin, Bax, Bcl-2-associated X protein, MDA, malondialdehyde, ROS, reactive oxygen species, Bcl-2, B-cell lymphoma-2, SOD, superoxide dismutase, CAT, catalase, CK, creatine kinase, CK-MB, creatine kinase-MB isoenzyme, LDH, lactate dehydrogenase, NLRP3, NOD-like receptor family pyrin domain containing 3, IL-1β, interleukin-1 beta, IL-18, interleukin-18.

The markers of miRNAs that are regulated through drug intervention are marked in bold.

### 3.4 Regulation of inflammation responses and calcium homeostasis

Inflammation is the immune system's response to harmful stimuli, which can be infectious or non-infectious (107). Several investigations have discussed between inflammation and heart failure, providing comprehensive summaries of various types of inflammation and their underlying mechanism (108). An imbalance between pro- and anti-inflammatory cytokines is a key feature of the HF state (109). High levels of pro-inflammatory cytokines are linked to elevated adverse outcomes for HF (110). Neutrophils release most of these cytokines at the cell level. These cytokines cause cardiomyocyte apoptosis and matrix metalloproteinase activation at the cellular level, which results in cardiac remodeling and changes in heart function (111). Numerous pro- and anti-inflammatory cytokines, including IL-1, IL-6, IL-8, IL-18, IL-1RA, and IL-33, have been demonstrated to be important in HF (112). Despite extensive research, the precise mechanisms that connect inflammation to heart failure remain uncertain, indicating a need for further investigation in this area (108).

Variations in cardiomyocyte calcium concentration impact the heart's contractility, which in turn alters heart function. All phases of the cardiac cycle are essentially regulated by Ca^2+^ homeostasis (113, 114). Myocardial contractility is directly dictated by the concentration of Ca^2+^ in the cytosol (115). In cardiac excitation-contraction coupling, the level of calcium in the cell's cytosol rises about tenfold. This increase occurs as extracellular Ca^2+^ enters the cardiomyocyte through the calcium channel CaV1.2 located in the cardiac cell membrane, subsequently stimulating the release of additional Ca^2+^ from the sarcoplasmic reticulum membranes (116). Relaxation in cardiac muscle occurs when Ca^2+^ is transported back into the sarcoplasmic reticulum (SR) by the SERCA2a pump (117). In cases of heart failure, there is an overaccumulation of cytosolic calcium caused by either decreased calcium efflux from the cytosol or increased calcium entry from the extracellular fluid and SR (118).

Numerous miRNAs can target and modulate inflammatory factor expression, lower inflammatory reactions, and improve the prognosis of HF. Some miRNAs can also balance calcium homeostasis and maintain myocardial electrophysiological balance. The HF mouse model established utilizing TAC and HL-1 cells was induced with H_2_O_2_ for *in vitro* and *in vivo* investigations. Overexpressing miR-17-5p was shown to lower NLRP3, Cleaved-Caspase-1, and GSDMD-N levels as well as prevent cardiomyocyte pyroptosis and inflammation (119). Gu et al., established a CHF rat model through coronary artery ligation and found that emodin could significantly up-regulate the expression of miR-26b-5p, target and inhibit PTEN, reduce ANP, BNP, IL-6, and TNF-α levels, and inhibit inflammation and myocardial injury (120).

Research demonstrates that patients with acute heart failure (AHF) have significantly lower levels of miR-106a-5p in their plasma compared to their healthy counterparts, and that these levels are negatively correlated with NT-proBNP and hs-CRP. Notably, AHF patients with a poor prognosis had markedly lower plasma miR-106a-5p levels than those with good prognoses (121). Xu et al. (122) prepared a mouse model of post-myocardial infarction heart failure (MI-HF) by coronary ligation and found that the NuanXin Capsule (NXC) could increase miR-126-3p, target and inhibit SPRED1, activate the VEGF-C pathway, and reduce the levels of type I collagen, type III collagen, IL-6, and TNF-α. Increase the expressions of VEGF-C, VEGFR3, Akt, and ERK1/2, promote angiogenesis of cardiac lymphatic vessels, and improve inflammatory responses and myocardial fibrosis. ZHAO (123) found that miR-155 was decreased in the ISO-induced HF rat model. Thus, overexpressing miR-155 can inhibit the levels of TGF-β, IL-4, and LPS, alleviate inflammatory responses, and myocardial fibrosis.

The investigation discovered that in the HF rat model created by left coronary artery ligation, the expression of miR-208a was lowered. Overexpression of miR-208a could target and inhibit β-catenin, reduce MMPs and IL-6 levels, and decrease myocardial cell apoptosis, inflammatory response, and extracellular matrix deposition (124). *In vitro* and *in vivo* experiments found that miR-214-3p was elevated in the HF rat model prepared by ISO injection. Inhibiting miR-214-3p could target and restore SERCA2a expression, enhance the calcium uptake function of the sarcoplasmic reticulum, and improve calcium homeostasis (125). ISO treatment triggered changes in H9c2 heart muscle cells, including markedly lowered miR-200b-3p levels. MiR-200b-3p overexpression can target and inhibit ZEB1, lower TNF-α, IL-6, and IL-β1 levels, and prevent cardiomyocyte myocardial cell injury and inflammation (126).

By reducing inflammatory factors and restoring myocardial calcium homeostasis, miRNAs reduce cardiac remodeling and enhance cardiac function, offering a novel targeted intervention strategy for the management of HF. The mechanisms and targets of miRNA regulation of inflammatory response and calcium homeostasis are outlined in Table 4 (119–126).

**Table 4:** The mechanism and targets of miRNA in regulating inflammation responses and calcium homeostasis.

Models and intervention methods	miRNAs	Regulatory indicators	Mechanism of action	References
TAC was used to prepare HF mouse models and H_2_O_2_-induced HL-1 cells	miR-17-5p↑	NLRP3, Cleaved-Caspase-1, GSDMD-N↓	Inhibit pyroptosis and inflammation of myocardial cells	(119)
Coronary artery ligation was used to establish a CHF rat model, and emodin was administered	**miR-26b-5p↑**	ANP, BNP, IL-6, TNF-α↓	Target PTEN to inhibit inflammation and myocardial damage	(120)
Patients with AHF	miR-106a-5p↑	NT-proBNP, hs-CRP↓	Reduce inflammatory responses	(121)
The MI-HF mouse model was prepared by coronary ligation and treated with NXC	**miR-126-3p↑**	COLI, COLIII, IL-6, TNF-α↓	Target SPRED1 to reduce inflammatory responses and improve myocardial fibrosis.	(122)
		VEGF-C, VEGFR3, Akt, ERK1/2↑		
The HF rat model was established by injecting ISO	miR-155↑	TGF-β, IL-4, LPS↓	Reduce inflammatory responses and myocardial fibrosis	(123)
The rat model of HF was prepared by ligation of the left coronary artery	miR-208a↑	β-catenin, MMPs, IL-6↓	Reduce myocardial cell apoptosis, inflammatory response, and extracellular matrix deposition	(124)
The HF rat model was established by injecting ISO	miR-214-3p↓	SERCA2a↑	Target SERCA2a to improve calcium homeostasis	(125)
ISO-induced H9c2 cardiomyocytes	miR-200b-3p↑	IL-6, IL-β1, TNF-α↓	Inhibit the inflammatory response of myocardial cells and myocardial cell damage	(126)

TAC, transverse aortic constriction, HF, heart failure, NLRP3, NOD-like receptor family pyrin domain containing 3, CHF, chronic heart failure, ANP, atrial natriuretic peptide, BNP, B-type natriuretic peptide, IL-6, interleukin-6, TNF-α, tumor necrosis factor-alpha, AHF, acute heart failure, hs-CRP, high-sensitivity C-reactive Protein, COLI, collagen type I, COLIII, collagen type III, Akt, protein kinase B, ISO, isoproterenol, TGF-β, transforming growth factor-beta, LPS, lipopolysaccharide, MMPs, matrix metalloproteinases.

The markers of miRNAs that are regulated through drug intervention are marked in bold.

## 4 Discussion

Over the past few years, it has been revealed that miRNA is a major regulator of the onset and progression of HF. Extensive research indicates that miRNAs participate in the regulatory network of HF by controlling various pathophysiological processes at the post-transcriptional level, including fibrosis, autophagy, apoptosis, inflammatory response, and cardiomyocyte energy metabolism. Although the mechanism of action of miRNAs in HF has made initial progress, there are still many challenges and limitations, which are briefly discussed as follows.

### 4.1 Complex mechanism of action and system analysis strategies

MiRNAs interact with a wide array of target genes, and their regulatory pathways often overlap significantly. In different cells or at different phases of the disease's progression, the same molecule may exhibit opposing effects. For instance, miR-155 exhibits a dual role of “protection” and “injury” in heart failure, which essentially reflects its context-dependent functionality (58, 85, 123). This discrepancy mainly arises from the interplay of three factors. First, differences in disease models and pathological mechanisms influence the regulatory direction of miR-155: in pressure overload models, endogenous downregulation of miR-155 occurs, and its overexpression can attenuate cardiomyocyte apoptosis by inhibiting HIF-1α; whereas in isoproterenol-induced hypertrophic heart failure, aberrant upregulation of miR-155 activates the AKT/CREB pathway to promote cardiac hypertrophy, making its inhibition protective instead. Second, target cell type is critical for functional divergence: in cardiomyocytes, miR-155 primarily regulates apoptosis and hypertrophic signaling; while in macrophages, it suppresses TGF-β secretion to block M2 polarization, thereby indirectly alleviating myocardial fibrosis. Finally, expression levels and spatiotemporal dynamics affect its net effect: under endogenous low expression, supplementation of miR-155 restores protective signals; however, under pathological conditions with abnormal high expression, inhibition is required to block detrimental pathways. Therefore, miR-155 is not a simple “pro-injury” or “anti-injury” molecule, but rather a pivotal regulatory node in the cardiomyocyte-immune interaction network, whose ultimate effect depends on the priority and relative abundance of target gene networks within specific pathological microenvironments, posing strategic demands for clinical intervention that must be tailored to timing, disease subtype, and cell type.

The mechanism of action is complex, and it is difficult to identify the intervention nodes. In the future, methods such as single-cell miRNA-seq, spatial transcriptome, and high-throughput screening should be utilized to construct a three-dimensional dynamic map. This information can be used to elucidate its mechanism of action and regulatory targets, and promote precise diagnosis and effective treatment. Notably, these technologies are no longer merely conceptual. Recent integrated single-cell and spatial transcriptomic analyses of pressure-overloaded mouse hearts have already begun to resolve the spatiotemporal crosstalk between cardiomyocytes and microenvironmental cells during adverse remodeling, revealing cell-type-specific and region-specific expression patterns that bulk analyses obscure (127). High-throughput screening platforms using human iPSC-derived cardiomyocytes (hiPSC-CMs) have already yielded functional miRNA mimics, such as hsa-miR-590-3p and hsa-miR-199a-3p, that promote cardiomyocyte proliferation, demonstrating the feasibility of using patient-derived cells and microfluidic chips to rapidly identify and optimize oligonucleotide sequences (128). Moving forward, combining scRNA-seq-derived cell atlases with spatial transcriptomics and hiPSC-based phenotypic screening offers a concrete pathway to resolve cell-type-specific miRNA functions and prioritize intervention nodes.

### 4.2 Insufficient clinical evidence and transformation pathways

We have discovered that there is limited large-sample, multi-center, longitudinal cohort validation, a gap in marker thresholds and standardized procedures, and that research in this area primarily concentrates on animal and cell experiments. It is hoped that relevant clinical trials can be carried out in the future, and peripheral blood, biopsy, imaging, and follow-up data can be collected simultaneously. Mirna-multi-omics—molecular phenotypic-related data can be integrated to transform the differential expression profile into early diagnosis and prognosis evaluation markers that can be applied in clinical practice.

It is important to distinguish between claims that are well supported and those that remain speculative. The diagnostic and prognostic value of circulating miRNA panels has been consistently demonstrated across multiple cohorts, with multi-marker strategies outperforming single miRNAs and retaining predictive value even after adjustment for natriuretic peptides and clinical covariates (129). Similarly, miRNA dynamics have been shown to track reverse remodeling in response to sacubitril/valsartan and cardiac resynchronization therapy, suggesting their utility as mechanistically grounded monitors of therapeutic response (129). These findings are supported by established reference ranges in whole blood and reproducible associations with NYHA class, LVEF, and hospitalization rates. By contrast, the clinical utility of miRNAs as standalone replacements for natriuretic peptides or imaging remains speculative; pre-analytical variation, platform heterogeneity, and the lack of standardized normalization procedures continue to hinder cross-study comparability (129). Moreover, sex-specific and HFpEF-specific data are only beginning to emerge, and many cohorts remain small, single-center, and enriched for HFrEF. Thus, while the biomarker potential is promising, its translation into routine laboratory practice requires rigorous, prospective multicenter validation.

### 4.3 One-way research perspective and network intervention

Most studies adopt a one-way perspective of “miRNA → mRNA”, ignoring the reverse regulation exerted by lncRNA, DNA methylation, and RNA editing, resulting in one-sided intervention points and easy compensatory rebound. Subsequent research can integrate the epigenetic-transcriptional-metabolic axis, analyze the positive feedback loop of miRNA and NAD⁺ -sirtuins, acetyl-CoA-histone acetylation, design the “metabolism-epigenetic -miRNA” triple therapy, and improve clinical efficacy.

Furthermore, it is essential to place miRNA-based strategies within the broader landscape of competing RNA therapeutics. Long non-coding RNAs (lncRNAs) and circular RNAs (circRNAs) are actively investigated in HF and offer both complementary and rival approaches. LncRNAs such as MALAT1 and SRA1 function as competing endogenous RNAs (ceRNAs) that sponge miRNAs, thereby derepressing pro-fibrotic targets and modulating hypertrophic signaling (130). CircRNAs, by contrast, exhibit remarkable stability and tissue-specific expression, making them attractive as both biomarkers and therapeutic targets. For example, circHIPK3 promotes cardiac fibrosis by sponging miR-29b-3p and derepressing collagen genes, whereas circPan3 exerts protective effects by sponging miR-320-3p to upregulate HSP20 (129). These lncRNA-miRNA-mRNA and circRNA-miRNA-mRNA axes constitute multilayered regulatory networks that dynamically shape miRNA availability. Consequently, therapeutic strategies that solely target a single miRNA risk being offset by compensatory changes in the ceRNA network. An integrated approach—simultaneously modulating miRNAs and their upstream lncRNA/circRNA sponges, or leveraging the superior stability of circRNAs as delivery scaffolds—may yield more durable clinical outcomes than miRNA monotherapy.

### 4.4 Individual differences and scientific medicine

The miRNA profiles of ischemic heart failure, valvular heart failure, and hereditary heart failure are different. Existing reports collectively categorize these conditions as heart failure, although they cannot be accurately differentiated. Subsequently, individual heart failure models can be replicated *in vitro* using the patient's IPSC-cardiomyocytes and high-throughput microfluidic chips to rapidly screen the optimal oligonucleotide sequences and doses, forming a replicable personalized medical paradigm. Furthermore, an ethics-payment-regulatory framework can be established. This setup will help miRNA therapy shift from lab work to real clinical care.

This vision is increasingly grounded in ongoing technological convergence. High-throughput miRNA mimic and inhibitor screens in hiPSC-CMs have already identified active candidates for cardiac regeneration (128). When coupled with single-cell transcriptomic atlases of human HF, these platforms can validate whether a candidate miRNA modulator produces the predicted cell-type-specific effect before advancing to clinical trials. Establishing an ethics-payment-regulatory framework around such personalized screening pipelines will be critical to translating these laboratory advances into reimbursable, standardized clinical workflows. Furthermore, conducting in-depth research on the mechanism and efficacy of traditional Chinese medicine in regulating miRNAs, and promoting the integration of traditional and Western medicine in the treatment of heart failure, is expected to enhance clinical efficacy (131–134).

In conclusion, miRNAs, as important regulatory factors of heart failure, have broad prospects for mechanism research and potential for clinical transformation. As multi-omics technology grows and the idea of scientific medicine develops, miRNAs stand to serve as new treatment targets. These tools will aid early detection, outcome prediction, and custom care plans for HF. However, realizing this potential requires moving beyond cataloging differential expression: future work must critically weigh the strength of existing evidence, situate miRNAs within the competitive RNA therapeutic landscape, and anchor emerging technologies to concrete, ongoing research programs rather than treating them as abstract aspirations.
